# My Ship at Sea (Poetry)

**Published:** 1881-10

**Authors:** 


### MY SHIP AT SEA.

How many ships I've sent to sea—
Buoyant with hope and full of glee,
How few returned to me!
Ships that I've freighted with my all;
Drifted away beyond recall,
But storms will rise and storms will fall,
And ships go down at sea.

How oft, with sails all golden—bright
With sunlight—they have passed from sight
While from the shore have I
Kept watch with eager eye,
Until my ship had floated by;
Until with sails all proudly set,
Just where the earth with heaven met,
They vanished—while I lingered yet.

But storms will come, and winds will blow—
My ships are driven to and fro—
And some go down at sea!
And some mere wrecks, from out the past—
Mere hull, and spar, and broken mast,
With all their treasures overcast,
Float back to me.

And then I sigh o'er what I've lost;
Weep o'er my life so tempest tost—
So cheerless—and so drear!
Why trust frail barks unto the sea?
What bring they back but grief to me?
But grief, and pain, and misery,
To rend my soul with fear!

These shattered wrecks the cruel sea
Casts on the shore to torture me
Are filled with phantoms dread!
Phantoms of all I've lost before—
Of hopes and joys dead in the yore;
Of hopes and loves that come no more;
And with these dead from unknown shore
Come other dead to make me sore—
The cruel, living dead!

But still, forgetting all my pain,
My barks I launch upon the main,
To cross the heaving sea,
Hoping that when all storms are past,
Some sunny port I'll reach at last,
To find with joy, all anchored fast,
My ships, awaiting me!

